# Periodontal Patients’ Perceptions and Knowledge of Dental Implants—A Questionnaire Study

**DOI:** 10.3390/jcm13164859

**Published:** 2024-08-17

**Authors:** Ewa Dolińska, Anna Węglarz, Weronika Jaroma, Gabriela Kornowska, Zuzanna Zapaśnik, Patrycja Włodarczyk, Jakub Wawryniuk, Małgorzata Pietruska

**Affiliations:** 1 Department of Periodontal and Oral Mucosa Diseases, Medical University of Bialystok, ul. Waszyngtona 13, 15-269 Bialystok, Poland; mpietruska@wp.pl; 2 Student’s Research Group at the Department of Periodontal and Oral Mucosa Diseases, Medical University of Bialystok, ul. Waszyngtona 13, 15-269 Bialystok, Poland; anna.aweglarz@gmail.com (A.W.); 40693@student.umb.edu.pl (W.J.); g.kornowska@gmail.com (G.K.); zapasnikzuzanna@gmail.com (Z.Z.); patiw222@gmail.com (P.W.); 40724@student.umb.edu.pl (J.W.)

**Keywords:** dental implant, implant surgery, questionnaire, tooth loss, periodontal disease, awareness

## Abstract

**Background:** Highly prevalent tooth loss is observed among populations around the world. To restore masticatory function and satisfactory aesthetics, missing teeth must be replaced. Dental implants are increasingly used for this purpose. This study aimed to assess periodontal patients’ knowledge and attitudes towards dental implants. **Methods:** 467 anonymous questionnaires of periodontal patients were analyzed. The population participants were divided according to gender, age, education and place of residence. In the statistical analysis, the chi-squared test of independence was used (*p* < 0.05). The main questions addressed patients’ knowledge about dental implants and the factors influencing their decision to undergo implantation. **Results:** The majority of periodontal patients were aware of dental implants and the importance of oral hygiene in their maintenance. However, the population studied had many knowledge deficits, especially on the technical and biological aspects of implants. The lack of knowledge about peri-implantitis was surprising in the group seeking professional periodontal care. Patients obtained information mainly from the internet and from family and friends, with their dentists being the third source. Good functional and aesthetic outcomes would encourage them to undergo the implantation procedure, and high cost and the possibility of complications were the most discouraging factors. **Conclusions:** Given the growing popularity of implant treatment, patients should be provided with evidence-based knowledge about indications and possible contraindications to implants to make informed decisions.

## 1. Introduction

Periodontitis is one of the main infectious diseases affecting the stomatognathic system, primarily in adults [1]. This problem affects 743 million people worldwide, approximately 11% of the population [2]. The course of periodontitis is influenced by various factors, but the disease is mainly caused by the accumulation of dental plaque containing a wide range of micro-organisms that cause inflammation leading to the destruction of the tissues surrounding the tooth—periodontal ligament and alveolar bone [3]. As a result, teeth are lost, worsening the patients’ quality of life.

Highly prevalent tooth loss is observed among populations worldwide, despite dynamic development of dentistry and progress in the field of prevention [4]. The main indications for dental extractions are still caries and periodontal diseases. Additionally, there are other causes that include tooth injuries, complications arising from endodontic treatment, orthodontic and prosthetic reasons, root fractures and complicated tooth eruptions [5]. Moreover, a relationship was shown between older age, lower socioeconomic status, lower level of patient education and an increased rate of tooth loss [6]. Loss of teeth and eventual edentulism results in chewing disorders which can carry a risk of malnutrition [7,8]. Other problems include phonation, esthetics and psychological aspects like sadness, shame and loss of self-esteem [9,10].

To restore masticatory function and satisfactory aesthetics, missing teeth must be replaced. Dental implants are increasingly used for this purpose [11]. The possibilities of restoring tooth deficiencies offered by dental implantology are enormous. Implants are used to replace single missing teeth, both in the aesthetic zone [12,13] and in the posterior area [14,15]. They are utilized to reconstruct completely or partially toothless alveolar ridges [16,17]. It is possible to make fixed or removable implant-supported prostheses [16]. Provisional narrow-diameter implants can support temporary dentures as well [18]. The clinical protocol for the placement of dental implants has changed over the years, now focusing on accelerated osseointegration and aesthetics. Previously, implants were placed into healed ridges. The use of implants with rough surfaces has accelerated osseointegration and decreased the required healing time. In optimal conditions, immediate loading is acceptable [19]. The development of predictable augmentation techniques and the introduction of novel implant surfaces has resulted in improving treatment outcomes, even in priority aesthetic regions [20]. Patients’ perceptions are becoming more and more significant in evaluating treatment outcomes in implant dentistry, which was noted at the 8th European Federation of Periodontology Consensus Conference [21].

Replacing missing teeth or even the entire dental arch with implant-supported restorations is a predictable treatment modality. It is becoming more and more popular because it preserves adjacent teeth and bone [22]. In the United States, there has been significant increase in the prevalence of dental implants among adults from 0.7% in 1999 to 2000 to 5.6% in 2015 to 2016. It is expected that it may reach up to 23% by the year 2026 [11]. Despite its advantages, patients often hesitate toward implant prosthetic treatment. The most important discouraging factors can be costs, treatment timespan, awareness and cultural attitudes, and the possibility of complications [23]. Identifying constraints could help to improve the acceptance rate and understand the reluctance of patients to undergo implant treatment. This is particularly important in the group of patients with periodontitis, as they are often potential patients for implantation. At the same time, it should be remembered that periodontitis is a multifactorial inflammatory disease. Systemic and local risk factors will also affect implants, which may lead to inflammatory complications. To date, several surveys have been carried out on patient knowledge of implants in different populations worldwide [24,25,26,27,28,29,30]. But to the best of our knowledge, this is the first survey of this specific patient group. 

Considering the above, the aim of the present study was to assess periodontal patients’ knowledge and their attitudes towards dental implants. The study also aimed to assess factors encouraging and discouraging implantation.

## 2. Materials and Methods

### 2.1. General Methodology and Questionnaire

An anonymous questionnaire was collected from 508 periodontal patients of the Outpatient Clinic of the Department of Periodontal and Oral Mucosa Diseases at the Medical University of Bialystok, Poland, in the period from 17 February 2023 to 31 May 2024. The response rate was 23.5%. According to other similar studies, 508 is a suitable sample size for analysis [24,26,27,28,29]. The studied population consisted of regular outpatients who were referred to the clinic for periodontal problems. The survey explored patients’ knowledge and opinions on dental implants. It was composed of closed-ended questions based on three sections: sociodemographic data, knowledge and attitude, and encouraging and discouraging factors. To analyze the population, participants were divided according to gender, age, education and place of residence. The exclusion criteria were being aged under 18 years old and already having dental implants. Patients who reported having dental implants in the questionnaire were excluded from the main analysis due to their personal experience and greater knowledge of implantation.

The main questions included in the questionnaire were connected with:

- patients’ attitudes to missing teeth and sources of knowledge about dental implants;
- patients’ knowledge about dental implants and understanding of the implantation process;
- knowledge about peri-implantitis and other treatment implications;
- factors encouraging and discouraging patients to undergo implantation.

The research was conducted in accordance with the Declaration of Helsinki and approved by the local bioethics committee (APK.002.111.2023, approval date 16 February 2023). Subjects who decided to fill in the anonymous questionnaire voluntarily were considered to have given their consent to participate in the study. They were informed that the data would be used for collective analysis and that they had the option to withdraw at every stage of filling in the form.

### 2.2. Statistical Analysis

At first, the patients were divided into two groups based on whether they answered that they had dental implants embedded or not. The first group (patients after implantation) were excluded from the main analyses because they had personal experience with dental implants.

For descriptive statistics, at first the number and percentage of replies to each question were counted. Answers to each question were analyzed according to the gender and age of the participants as well education and participants’ place of residence. If the question assessed knowledge, the number of correct answers was analyzed. If the number of correct answers did not exceed 80%, a conclusion of insufficient knowledge was drawn. Because some questionnaire variables were incomplete, the total numbers for some of the data collected in the questionnaire differ, and the percentages in the tables are presented as the percentage of valid items, not of the total.

In the statistical analysis, the chi-squared test of independence was used to check the relationship between qualitative characteristics. Statistical significance was established at *p* < 0.05. Calculations were made using Statistica 13.3 (TIBCO Software Inc., Palo Alto, CA, USA).

## 3. Results

### 3.1. Description of the Group Studied

The written survey was filled in by 508 periodontal patients of the Outpatient Clinic of the Department of Periodontal and Oral Mucosa Diseases at the Medical University of Bialystok, Poland.

Because 41 patients declared already having an implant, their questionnaire forms were excluded. That is why the study is based on the answers of 467 participants, out of whom 318 were women, 142 were men, and 7 did not give their gender. Patients were divided into the following age groups: 18–30 years, 31–50 years, 51–70 years, and 71 years and above. Three people did not give their age. Participants also answered a question about their education: 2.4% declared primary school education, 39.5% declared secondary school education, 10.7% had incomplete higher education and 45.8% higher education. Two participants described their education as other. Another factor that was taken into account in the analysis was place of residence: 19.1% were rural area residents, 22.4% lived in cities not bigger than 50,000 inhabitants and 54.6% in cities exceeding 50,000 inhabitants; 3.9% people did not provide their place of residence. To analyze the group, the patients were also asked if they had missing teeth. Most of participants (337) had missing teeth, and 126 declared full arches; 4 people did not provide an answer. Participants’ general characteristics are presented in Table 1. The answers to the survey questions in numerical and percentage forms in the analyzed population are presented in Table 2.

### 3.2. Patients’ Attitudes to Missing Teeth and Sources of Knowledge about Dental Implants

Summary:

An overwhelming majority of the patients considered the replacement of missing teeth to be very important and most had heard about dental implants. The main sources of information for participants were the internet, friends and family, and their dentists. However, most of patients wanted to know more about dental implants.

Details:

An overwhelming majority of the patients felt that replacing missing teeth is very important (95.7%), and most of them (93.1%) had heard about dental implants. However, 60.7% of participants admitted that they had heard rather little. Whether the patient had heard about dental implants depended on gender (*p* = 0.0015; most answers (77%) that they had heard a lot were given by women), age (*p* = 0.003), education (*p* = 0.03; the higher the education level, the fewer patients had not heard of implants) and having missing teeth (*p* = 0.04; almost 10% of patients who had full arches had not heard about dental implants). The main sources of knowledge for participants were the internet (42.6%), friends and family (37.6%), a dentist (32.9%), radio and television (19.7%), newspapers and magazines (18.6%) and other (4.8%). The younger the person, the more often the answer “other” was given (*p* = 0.01), and people with higher education used the internet more often as a source of knowledge (*p* = 0.002).

Most patients wanted to know more about dental implants (62.8%). The lower the education level, the lower the desire to deepen their knowledge (*p* = 0.04). Only 35% of respondents had heard direct opinions from somebody with a dental implant (most of these answers were given by women—76% (*p* = 0.006)). Patients who had missing teeth wanted to know more about implants (*p* = 0.01), and they had heard direct opinions more often than patients with all their teeth (*p* = 0.006). Most direct opinions came from friends (57%) and family (35.8%) but also from the internet (11%) and television (7.4%). In 90.3% of cases, the direct opinions were positive.

### 3.3. Patients’ Knowledge about Dental Implants

Summary:

The majority of patients knew that implants are inserted into the bone and should be cared for like teeth. Only slightly more than half were aware that the procedure can fail. Knowledge deficits also concerned the impact of periodontitis or the occurrence of peri-implantitis. Most patients had also not heard of the need for bone augmentation. Many patients did not know what material implants are made of.

Details:

To assess patients’ knowledge, questions concerning implant insertion and the material from which the implant is made were asked. In general, most answers relating to implant placement were correct: 79,6% stated that dental implants are embedded in the maxilla or mandible bone. However, almost 15% of participants thought that implants are placed in the gum, and 7% on adjacent teeth. There was a connection between education and incorrect answers to this question. Patients with primary education were more likely to answer incorrectly than those with higher education (*p* = 0.013). Taking into account the material of dental implants, the most frequent answer was “I don’t know”. Only 26.6% of the population studied chose titanium and 10.6% ceramics. In the subgroup analysis, women were more likely to choose titanium (78% answers, *p* = 0.02) and men ceramics (56% of “yes” answers *p* < 0.0001). The youngest participants more often chose titanium (*p* = 0.0002), as did patients with better education (*p* = 0.003).

Other knowledge questions concerned the course of implantation and possible contraindications. Almost half of the respondents did not know if implantation is suitable for every patient, and 74% of negative answers were given by women (*p* = 0.017). Awareness of possible implant failure was found in 50.8% participants, but 49.2% had not heard about implant failures. Only 36.8% of the population was conscious that periodontitis can be a problem in dental implant treatment. Younger patients were more likely to take periodontitis into consideration (*p* = 0.001). Over half of the people questioned had not heard about bone augmentation procedures. Women had heard about them significantly more often (*p* = 0.018), as had better educated participants (*p* = 0.015). A satisfactory level of knowledge of implant hygiene was noted: 92.7% of people answered that implants have to be maintained like natural teeth (95% women and 87% man; *p* = 0.001). In every age group, correct answers exceeded 80%, with the highest prevalence in the youngest group (*p* = 0.025). Education also influenced responses (*p* = 0.005). Only 67% of participants from the group with primary education answered that maintenance of implants should be the same as for teeth. In contrast, most periodontal patients had not heard about peri-implantitis (72%). There were 37% of women and 22% of man who were aware of peri-implantitis (*p* = 0.04). Education had also an impact (*p* = 0.02): the more extended their education, the more prevalent was knowledge about peri-implantitis. A thorough analysis of the answers to knowledge questions is summarized in Table 3.

### 3.4. Encouraging and Discouraging Factors to Implantation

Summary

**Figure 1:**
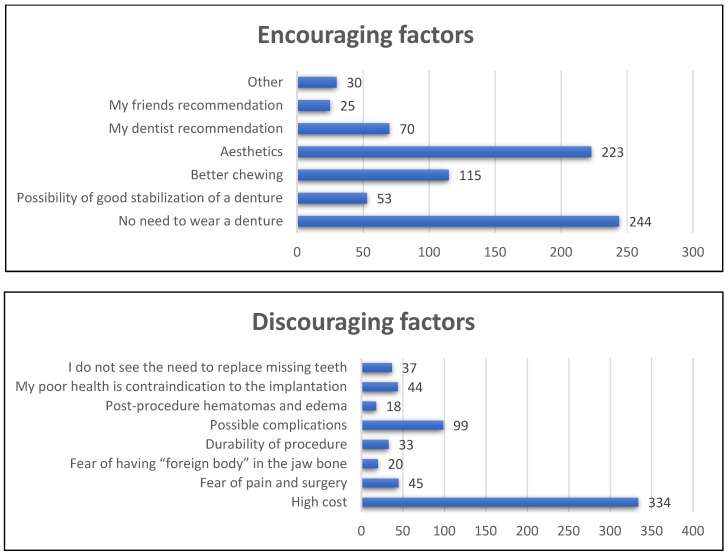
Encouraging and discouraging factors for implantation in general population studied (number of answers).

According to our study, the most encouraging factors for implantation are no need to wear dentures, aesthetic appearance and the possibility of better chewing. Among discouraging factors, the high cost of the procedure was the most frequently mentioned. The answers of the whole population are depicted in Figure 1.

Details

The most encouraging factors in favor of implantation according to our study are no need to wear a denture (54.1%), aesthetic appearance (49.4%) and the possibility of better chewing (25.5%).

Other benefits were chosen much less frequently and did not exceed the 16% threshold. The most motivating factor, which was avoiding removable dentures, was connected with the age (*p* = 0.02), gender (*p* = 0.005) and education of the respondents (*p* = 0.02). Periodontal patients with missing teeth more often paid attention to stabilization of a dental prosthesis (*p* = 0.02). For women, aesthetic appearance was more important than for men (*p* = 0.03), and men were more likely to listen to their dentist’s (*p* = 0.02) and friends’ opinions (*p* = 0.005).

Among discouraging factors, the high cost of the procedure was the most frequently mentioned (76.3%), followed by awareness of possible complications (22.7%), fear of pain, swelling and the surgical procedure itself (10.8%) and consciousness that overall health condition may not allow for implantation (10.1%). High financial costs were the least important to the youngest age group (*p* = 0.02) and to the group with missing teeth (*p* = 0.007). Significantly more men (*p* = 0.02) and people with full teeth (*p* = 0.007) did not see the need to replace missing teeth. The exact answers to questions about encouraging and discouraging factors are summarized in Table 4.

## 4. Discussion

The survey aimed to evaluate the knowledge of periodontitis patients about dental implants and their attitudes towards the implantation process. An overwhelming majority of participants in our study had heard of dental implants (93.1%). This is a higher percentage than in similar studies where the implant awareness rate was 77% in the USA [24], 70.7% in Norway [25] and 70.1% in Switzerland [26]. Patients were recruited to the study in the Outpatient Clinic of the Department of Periodontal and Oral Mucosa Diseases at the Medical University of Bialystok, Poland, so they were looking for professional periodontal care. They were aware of periodontal disease and the possibility of tooth loss. Probably the rate of knowledge about implants in the general Polish population might be lower, as in the other study concerning Polish citizens (75.6%) [27]. In other studies, the general population group was less informed about implants than patients presenting for implant screening [28]. Despite awareness of dental implants, most patients admitted that they know rather little about them. The main sources of information were the internet (42.6%), friends and family (37.6%) and dentists (32.9%). This is consistent with other surveys where the internet was the main data provider [24,27]. In Switzerland, dentists are mentioned more frequently than the internet and family and friends [29]. In Saudi Arabia, the major sources of data about implants were friends and families, followed by the internet and dentists [30]. Similarly, in Jordan, for 63.4% of people, relatives and friends were the main source of information, followed by dentists [31]. In Japan, information regarding dental implants came mainly from books and magazines, then friends and dental professionals [32]. All the studies confirm the very important role of dentists in informing patients and expanding knowledge about dental implants. Direct personal communication appears to be an important factor. Information from dental professionals is also the most reliable. In contrast, the internet and social media can give an impression that implant therapy is always possible and spread the idea of everlasting implants that do not require special attention and hygiene [33]. That is why the knowledge of patients is superficial and there is often misunderstanding of biological, technical and aesthetic considerations. In the society in general, there can be an impression that implantation is always possible and it is the best treatment option. Therefore, dental practitioners should spend more time explaining the possibilities and limitations of implant treatment. They are the most dependable source of information, and it is in their interest that the patient learns about all treatment options.

In our study, we assumed that a percentage of correct answers exceeding 80% indicates sufficient knowledge on a given topic. The highest percentage of correct answers concerned home hygiene of implants. Over 92% of the entire group answered that implants should be cared for like teeth. Correct answers were given by both women and men, and by patients from different age groups. The percentage of correct answers did not exceed 80% only in the least educated group. Such answers are not a surprise because the entire group was looking for professional periodontal care and most of them were aware than home hygiene comes first in controlling periodontal disease. A majority of periodontal patients (over 90%) was aware that inadequate oral hygiene may lead to periodontal disease, as was noted in a previous study by our group [34].

Almost 80% of respondents knew that implants are anchored in the bone. We noted a lack of knowledge in the oldest age group, the primary education group and in residents of villages. In the study of Nitschke et al., 80.5% of participants believed that implants are embedded in the bone [35], but in the study of Krupińska and Bogucki [27] only 60.4% of people answered correctly to this question. The level of knowledge is definitely lower when it comes to the material from which implants are made. In our survey, most patients chose “I don’t know”, and the answer “porcelain” (27.5%) was more prevalent than “titanium” (26.6%). In the age group 18–30 years old, 51% of people chose titanium, which was the highest percentage of answers relating to titanium as implant material. The lack of knowledge of dental implant material was noticed in other studies. Participants answered similarly (35% titanium and 33% ceramic) in the study of Nitschke [35]. Approximately 49% of males and 56% of females displayed their unawareness regarding materials used for dental implants in the study of Alkhaldi [30].

We noted many other knowledge deficits in the group studied. In the general population, only 34.2% of participants were aware that implant treatment is not for every patient, and only slightly over half knew that implant surgery may end in failure. Periodontitis was perceived as a problem in the context of implantation by 36.8% participants. The younger the participants, the higher percentage of them was aware that periodontitis may implicate implant treatment. Although the study concerned periodontitis patients, 53.9% were not sure if periodontitis affects implantation, and only 28% had heard about peri-implantitis. More women (31%) than men (22%) had heard about peri-implant diseases, but this is a very low proportion in the whole population studied. According to our statistical analysis, education had a strong impact on awareness of peri-implantitis. Peri-implantitis represents a growing public health problem worldwide. Because of the high prevalence of this disease (22% of patients having an implant) [36] and the associated consequences (implant and implant-supported prosthesis loss), patients should be better informed about the possibility of peri-implant diseases occurring.

Only 45.5% of patients had heard that, in some cases, bone augmentation for implant purpose is necessary. Better-educated groups were more aware of bone deficits being a problem. Knowledge of bone grafting in dentistry is not common in other populations as well. In Saudi Arabia, 68.2% of respondents had not heard about bone grafts and 64.2% did not know when it is indicated. Sixty-six percent would not decide to undergo augmentation even if it was needed [30], and in Japan 73% of study participants did not know that grafts were a part of treatment planning [32].

Polish periodontal patients were encouraged to choose dental implants mostly because it could eliminate the need to wear a denture (54%) and for aesthetic reasons (49%). Stabilization of dental prosthesis was important for people who already missed teeth. Aesthetics was significantly more important for women than men, and as many as 63% of people aged 18–30 years also selected aesthetics. The same results are seen in Switzerland, where female gender and young age were factors that correlated with higher value on aesthetic outcomes [29]. Dentist recommendations were motivating for men and people with higher education. Men would also rely on friends’ recommendations. Better chewing was crucial for only one quarter of respondents. Aesthetics and function were also the main reasons for implantation in other studies. For denture wearers in Jordan, function (55.7%) was more important than aesthetics (32.3%), and 47% of participants preferred having implants to avoid damaging adjacent teeth [31]. In patients in Japan, no need to wear a prosthesis and better mastication were the most common reasons to obtain implants, and aesthetics was in third place [32]. This is not surprising because in this study almost 30% of the respondents were over 60 years old.

Patients perceive implants differently than dental professionals. In a subjective assessment, implants were described as “expensive” (45%), “advanced” and “scary”, or even “painful” or “dangerous” [32]. This proves that patients have many concerns and that high financial cost is one of them. Also in our study, the most discouraging factor for implantation was the high cost of the procedure (76.3%). This is consistent with the results of other studies, where financial aspects were in first place [24,29,30,31,32,35,37]. However, in the study from Germany, patients would decide to have even expensive dental implants if they had to [35], and in the study from Jordan they would decide on implant if insurance would pay for the treatment [31]. In the study from Switzerland, patients desired the implantation to be performed by certified clinical specialists, and they indicated that they were ready to pay more for the same procedure in Switzerland than abroad [29]. Still, a tooth-supported dental prosthesis is more cost effective than an implant-supported single crown [38], and the high price of treatment is always a factor in the treatment decision. Other concerns of patients in our study were the possibility of complications (22.7%), fear of surgery and pain (10.3%) and feeling that their general health condition is inadequate for surgery (10.1%). Polish patients’ concerns are consisted with the worries of people from different countries, who fear surgery itself, long treatment times and complications [27,29,31,32,35].

The interesting thing is that patients who already have dental implants perceive them very positively. Research conducted in Germany and Switzerland aimed at assessing satisfaction in people 10 years after implantation. The evaluation in both studies was based on a survey and a visual analogue scale (VAS). Over 90% of patients were satisfied with the implant treatment, both from a functional and aesthetic point of view. Patients especially appreciated the chewing comfort, aesthetics and phonetic function. The vast majority of people had no problem maintaining hygiene around implants and would decide to undergo the implantation procedure again. Most participants would recommend the implantation procedure to friends and family and, surprisingly believed that the costs of the therapy were justified [39,40]. However, in the study by Wang et al., a slight deterioration in satisfaction was noted when moderate or severe inflammation of the peri-implant tissues occurred [40]. This is confirmed by a study from Thailand, where a similar decrease in contentedness was observed in patients who experienced postoperative complications or developed peri-implantitis [41]. In the 2022 review, patients’ preoperative expectations significantly influenced the perception of treatment results. The implants improved the stabilization of the prostheses and the functions of the masticatory system, but in the overall assessment, the oral health-related quality of life was not higher than when using conventional prosthetic restorations. Aesthetics was satisfactory for most patients. However, clinicians’ aesthetic expectations were much higher and did not match patients’ perceptions. Financial efficiency concerned only those people who wanted to invest more in increased comfort of life [42]. This proves that in some cases expensive implant treatment does not meet the patients’ high expectations. This may be due to a lack of knowledge about the achievable goals of implant therapy and its biological and mechanical complications. This is why studies like ours are important. They allow us to assess patients’ knowledge and their attitudes towards dental implants, and thus plan information programs. Proper education explaining the procedure reduces the likelihood of misinterpretation. Therefore, it is necessary to create reliable sources of information and provide educational paths for dentists so that they can effectively inform patients about implant treatment. With an aging population, the demand for implants will increase, especially in patients with periodontal disease. Taking into consideration all the above reliable information is needed so that patients can trust implant therapy.

Our study is a broad analysis of periodontal patients’ knowledge and attitudes to dental implants. However, it has some limitations. One of them is a survey of periodontitis patients only. Their answers could differ from the answers of the entire Polish population, because periodontal patients are aware of the possibility of losing teeth and may be more interested in different options for replacing them. The other is that the respondents may not have been involved enough in completing the survey or may have misunderstand the questions. There was also a lack of opportunity to explore the questions in greater depth because the questionnaire had a closed nature and participants could only choose from given options. Another limitation is in maintaining the survey nature of the study. Combining survey responses with periodontal status would provide insight into the actual treatment needs of patients. Despite these questionnaire limitations, over 500 people were surveyed, which allowed for advanced analysis of the results.

As implants are increasingly used in dental treatment, future studies of patient knowledge and attitudes are needed. Such studies could look at comparing the knowledge of periodontal patients with that of the general population. The lack of knowledge confirms, also, the need for patient education. The need for such non-commercial campaigns and their effectiveness in assessing patients’ knowledge of implants could also be a topic for research.

## 5. Conclusions

This survey-based study has indicated that a majority of periodontitis patients were aware of dental implants and the importance of oral hygiene in their maintenance. However, the population studied had many knowledge deficits, especially on the technical and biological aspects of implants. The lack of knowledge about peri-implantitis was surprising in a group seeking professional periodontal care. Patients obtained information mainly from the internet and from family and friends, and their dentists were in third place. Good functional and aesthetic outcomes would encourage them to undergo the implantation procedure, and high cost and the possibility of complications were the most discouraging factors. Taking into account the growing popularity of implant treatment, patients should be provided with evidence-based knowledge about indications and possible contraindications to implantation to avoid misunderstandings and consciously make treatment decisions.

## Figures and Tables

**Table 1:** Demographic characteristics of the study population.

		*n*	%
total		467	100
age	18–30	171	9.2
	31–50	194	36.6
	51–70	196	41.5
	71 and above	56	12
	not stated	3	0.6
gender	female	318	68.1
	male	142	30.4
	not stated	7	1.5
education	primary	11	2.4
	secondary	184	39.4
	incomplete higher	50	10.7
	higher	214	45.8
	other	2	0.4
	not stated	6	1.3
place of residence	village	89	19.1
	small city	105	22.5
	city above 50,000	225	54.6
	not stated	18	3.8
missing teeth	yes	337	72.2
	no	126	27
	not stated	4	0.8

**Table 2:** Answers to survey questions in analyzed population. (Not every patient answered every question. In questions with multiple answers, patients could select more than one answer. That is why the total numbers for some of the data collected in the questionnaire differ).

Question	Possible Answers	Number of Answers	Percentage of Answers
Do you think that replacing missing teeth is important?	Yes	447	95.7%
	No	3	0.6%
	I don’t know	17	3.6%
Have you heard about dental implants?	Yes, a lot	151	32.4%
	Yes, a little	283	60.7%
	No	32	6.7%
If yes, how did you learn about implants?	Dentist	145	32.9%
	Family and friends	166	37.6%
	Newspapers and magazines	82	18.6%
	Radio and television	87	19.7%
	Internet	188	42.6%
	Other	21	4.8%
Where are the implants placed?	In the jaw bone	343	79.6%
	In the gum	64	14.8%
	In the adjacent teeth	30	7%
What material are implants made of?	Porcelain	125	27.5%
	Stainless steel	34	7.5%
	Titanium	121	26.6%
	Ceramics	48	10.6%
	I don’t know	197	43.4%
Is the implant procedure suitable for every patient?	Yes	88	19.17%
	No	157	34.2%
	I don’t know	214	46.6%
Did you know that implantation may end in a failure?	Yes	229	50.8%
	No	222	49.2%
Do you think that periodontitis is a contraindication to implantation?	Yes	168	36.8%
	No	42	9.2%
	I don’t know	246	53.9%
Did you know that often, before implantation, bone reconstruction is needed?	Yes	203	45.5%
	No	243	54.5%
Did you know that implants should be cared for like teeth?	Yes	423	92.7%
	No	32	7%
Did you hear about peri-implantitis?	Yes	127	28%
	No	327	72%
Do you want to know more about implants?	Yes	284	62.8%
	No	168	37.2%
Did you hear direct opinion from someone who has a dental implant?	Yes	160	35%
	No	296	65%
If yes, who was it?	Someone from my family	63	35.8%
	A friend	101	57%
	Someone from TV	13	7.4%
	Someone from the internet	21	12%
Was the opinion positive?	Yes	158	90.3%
	No	17	9.7%
Are you satisfied with your prosthetic restorations?	Yes, very satisfied	69	24.4%
	Yes, they are acceptable	142	50.2%
	No	72	25.4%
What would encourage you to have an implant?	No need to wear a denture	244	54.1%
	Possibility of good stabilization of a denture	53	11.8%
	Better chewing	115	25.5%
	Aesthetics	223	49.4%
	My dentist’s recommendation	70	15.5%
	My friends’ recommendation	25	5.5%
	Other	30	6.7%
What discourages you from having an implant?	High cost	334	76.3%
	Fear of pain and surgery	45	10.3%
	Fear of having a “foreign body” in the jaw bone	20	4.6%
	Durability of procedure	33	7.6%
	Possible complications	99	22.7%
	Post-procedure hematomas and edema	18	4.1%
	My poor health is a contraindication to implantation	44	10.1%
	I do not see the need to replace missing teeth	37	8.5%
Do you think that you will decide to have dental implants in the future?	Yes	119	26.2%
	No	67	14.8%
	I don’t know	268	59%

**Table 3:** Patients’ knowledge about implants and the implantation process. Patients were analyzed according to age, gender, education, place of residence and having missing teeth.

	**Age group**				**Chi-squared**	***p***
**Implants** **are** **embedded** **in the bone**	18–30 (*n* = 36)	31–50 (*n* = 127)	51–70 (*n* = 146)	≥71 (*n* = 32)	7.9	*p* = 0.05
	84%	79%	83%	65%		
	**Male** (*n* = 100)		**Female** (*n* = 237)		0.1	NS
	79%		80%			
	**Education**					
	primary	secondary	incomplete higher	higher	13	*p* = 0.01
	*n* = 6	*n* = 122	*n* = 35	*n* = 174		
	67%	73%	74%	87%		
	**Place of residence**					
	village	small city		city above 50,000	0.7	NS
	*n* = 61	*n* = 79		*n* = 190		
	76%	81%		80%		
	**Missing teeth** (*n* = 244)		**Full teeth** (*n* = 97)		0.003	NS
	80%		80%			
	**Age group**				**Chi-squared**	***p***
**Implants** **are** **made of** **titanium**	18–30 (*n* = 22)	31–50 (*n* = 33)	51–70 (*n* = 59)	≥71 (*n* = 7)	24.4	*p* = 0.00002
	51%	20%	32%	13%		
	**Male** (*n* = 26)		**Female** (*n* = 92)		5	*p* = 0.02
	19%		30%			
	**Education**					
	primary	secondary	incomplete higher	higher	15.7	*p* = 0.003
	*n* = 1	*n* = 33	*n* = 20	*n* = 66		
	10%	19%	41%	32%		
	**Place of residence**					
	village	small city		city above 50,000		
	*n* = 21	*n* = 33		*n* = 61	2	NS
	24%	32%		24.5%		
	**Missing teeth** (*n* = 21)		**Full teeth** (*n* = 8)		0.01	NS
	7%		7%			
	**Age group**				**Chi-squared**	***p***
**Implants** **are not for every** **patient**	18–30 (*n* = 20)	31–50 (*n* = 53)	51–70 (*n* = 67)	≥71 (*n* = 16)	8	NS
	48%	32%	35%	29%		
	**Male** (*n* = 41)		**Female** (*n* = 115)		8	*p* = 0.02
	29%		37%			
	**Education**					
	primary	secondary	incomplete higher	higher	2	NS
	*n* = 3	*n* = 65	*n* = 16	*n* = 69		
	30%	35%	32%	33%		
	**Place of residence**					
	village	small city		city above 50,000	7	NS
	*n* = 24	*n* = 29		*n* = 96		
	28%	28%		38%		
	**Missing teeth** (*n* = 110)		**Full teeth** (*n* = 46)		2	NS
	33%		37%			
	**Age group**				**Chi-squared**	***p***
**Implantation** **may end in failure**	18–30 (*n* = 22)	31–50 (*n* = 78)	51–70 (*n* = 103)	≥71 (*n* = 25)	2.7	NS
	52%	47%	55%	47%		
	**Male** (*n* = 67)		**Female** (*n* = 160)		0.4	NS
	49%		52%			
	**Education**					
	primary	secondary	incomplete higher	higher	9	NS
	*n* = 2	*n* = 80	*n* = 28	*n* = 113		
	22%	45%	56%	55%		
	**Place of residence**					
	village	small city		city above 50,000	4	NS
	*n* = 35	*n* = 51		*n* = 134		
	41%	50.5%		54%		
	**Missing teeth** (*n* = 168)		**Full teeth** (*n* = 60)		0.2	NS
	52%		49%			
	**Age group**				**Chi-squared**	***p***
**Periodontitis** **may** **implicate** **implant treatment**	18–30 (*n* = 30)	31–50 (*n* = 62)	51–70 (*n* = 65)	≥71 (*n* = 10)	34	*p* = 0.00001
	71%	37%	35%	18.5%		
	**Male** (*n* = 45)		**Female** (*n* = 120)		1.8	NS
	32%		38.5%			
	**Education**					
	primary	secondary	incomplete higher	higher	11	NS
	*n* = 3	*n* = 62	*n* = 23	*n* = 79		
	30%	34%	46%	38%		
	**Place of residence**					
	village	small city		city above 50,000		
	*n* = 27	*n* = 39		*n* = 99	2	NS
	31%	38%		40%		
	**Missing teeth** (*n* = 108)		**Full teeth** (*n* = 60)		10	*p* = 0.006
	33%		48%			
	**Age group**				**Chi-squared**	***p***
**Implants ** **may need** **bone augmen-** **tation**	18–30 (*n* = 18)	31–50 (*n* = 77)	51–70 (*n* = 90)	≥71 (*n* = 17)	4	NS
	43%	46%	49.5%	33%		
	**Male** (*n* = 50)		**Female** (*n* = 150)		5.6	NS
	37%		49%			
	**Education**					
	primary	secondary	incomplete higher	higher	12	*p* = 0.01
	*n* = 1	*n* = 67	*n* = 24	*n* = 107		
	10%	39%	50%	52%		
	**Place of residence**					
	village	small city		city above 50,000		
	*n* = 34	*n* = 50		*n* = 115	2	NS
	40%	49%		47%		
	**Missing teeth** (*n* = 153)		**Full teeth** (*n* = 49)		3	NS
	48%		39%			
	**Age group**				**Chi-squared**	***p***
**Implants** **needs hygiene ** **like teeth**	18–30 (*n* = 40)	31–50 (*n* = 157)	51–70 (*n* = 178)	≥71 (*n* = 45)	9	*p* = 0.03
	95%	93%	95%	83%		
	**Male** (*n* = 120)		**Female** (*n* = 297)		10.5	*p* = 0.001
	87%		95.5%			
	**Education**					
	primary	secondary	incomplete higher	higher	15	*p* = 0.005
	*n* = 6	*n* = 162	*n* = 48	*n* = 199		
	66%	90%	96%	96%		
	**Place of residence**					
	village	small city		city above 50,000	4	NS
	*n* = 77	*n* = 98		*n* = 234		
	89%	96%		94%		
	**Missing teeth** (*n* = 303)		**Full teeth** (*n* = 116)		0.5	NS
	93%		94%			
	**Age group**				**Chi-squared**	***p***
**Did you hear about** **peri-implantitis?**	18–30 (*n* = 17)	31–50 (*n* = 42)	51–70 (*n* = 57)	≥71 (*n* = 10)	7	NS
	40.5%	25%	70%	18%		
	**Male** (*n* = 30)		**Female** (*n* = 96)		4	*p* = 0.04
	22%		31%			
	**Education**					
	primary	secondary	incomplete higher	higher	13	*p* = 002
	*n* = 0	*n* = 42	*n* = 15	*n* = 65		
	0%	23%	31%	31%		
	**Place of residence**					
	village	small city		city above 50,000	4	NS
	*n* = 27	*n* = 34		*n* = 60		
	31%	34%		24%		
	**Missing teeth** (*n* = 91)		**Full teeth** (*n* = 35)		0.04	NS
	27%		29%			

**Table 4:** Encouraging and discouraging factors for implantation in the population and subgroups analyzed.

	Total	Age					Gender			Education					Missing Teeth		
		18–30	31–50	51–70	71 and Above	*p*	Female	Male	*p*	Primary	Secondary	Incomplete Higher	Higher	*p*	Yes	No	*p*
*n*	467	171 9.2%	194 36.6%	196 41.5%	56 12%		318 68.1%	142 30.4%		11 2.4%	184 39.4%	50 10.7%	214 45.8%		337 72.2%	126 27%	
Encouraging factors																	
No need to wear prosthesis		18 42%	105 62%	98 54%	22 42%	*p* = 0.02	180 58%	60 44%	*p* = 0.005	2 22%	85 48%	30 60%	124 60%	*p* = 0.02	167 52%	75 60%	NS
Excellent stabilization of prosthesis		1 2%	16 9%	29 16%	7 13%	NS	35 12%	17 12%	NS	1 11%	23 13%	3 6%	24 12%	NS	45 14%	8 6%	*p* = 0.02
Better chewing		14 32%	31 18%	53 29%	16 31%	NS	80 26%	34 25%	NS	1 11%	51 29%	7 14%	54 26%	NS	80 25%	34 27%	NS
Aesthetic appearance		27 63%	86 51%	84 46%	24 46%	NS	163 53%	57 42%	*p* = 0.03	5 56%	76 43%	22 44%	114 55%	NS	155 45%	67 54%	NS
Dentist’s recommendation		3 7%	28 17%	33 18%	5 10%	NS	40 13%	29 21%	*p* = 0.02	1 11%	19 11%	3 6%	47 23%	*p* = 0.005	50 15%	20 16%	NS
Friends’ recommendation		2 5%	6 4%	12 7%	5 10%	NS	11 4%	14 10%	*p* = 0.005	1 11%	9 5%	0 0%	15 7%	NS	16 5%	9 7%	NS
Others		5 12%	8 5%	13 7%	4 8%	NS	17 6%	12 8%	NS	2 22%	17 10%	1 5%	10 5%	NS	19 6%	11 9%	NS
Discouraging factors																	
High cost		24 57%	127 78%	144 80%	37 76%	*p* = 0.02	236 78%	93 73%	NS	7 78%	138 80%	36 77%	147 73%	NS	255 80%	76 67%	*p* = 0.007
Fear of pain and surgery		6 14%	11 7%	22 12%	6 12%	NS	32 11%	12 10%	NS	1 11%	20 12%	2 4%	21 10%	NS	36 11%	9 8%	NS
Fear of foreign body		3 7%	7 4%	7 4%	3 6%	NS	10 3%	9 7%	NS	1 11%	7 4%	1 2%	11 5%	NS	13 4%	7 6%	NS
Long duration of treatment		5 12%	9 6%	15 8%	4 8%	NS	22 7%	11 9%	NS	0 0%	12 7%	1 2%	19 9%	NS	23 7%	10 9%	NS
Medical complications		15 36%	30 19%	43 24%	10 20%	NS	78 26%	20 16%	*p* = 0.02	1 11%	30 17%	15 31%	51 25%	NS	70 22%	29 26%	NS
Hematomas and swelling		1 2%	4 2%	8 4%	5 10%	NS	12 4%	6 5%	NS	2 22%	8 5%	2 4%	6 3%	NS	15 5%	3 3%	NS
My poor health is a contraindication		5 12%	11 7%	20 11%	7 14%	NS	31 10%	12 10%	NS	3 33%	20 12%	5 11%	15 7%	NS	32 10%	11 10%	NS
No need to restore missing teeth		1 2%	20 12%	12 7%	4 8%	NS	20 7%	17 13%	*p* = 0.02	0 0%	13 8%	1 2%	23 11%	NS	19 6%	17 15%	*p* = 0.002

## Data Availability

The datasets used and/or analyzed during the current study are available from the corresponding author on reasonable request.
